# Role of genetic and electrolyte abnormalities in prolonged QTc interval and sudden cardiac death in end-stage renal disease patients

**DOI:** 10.1371/journal.pone.0200756

**Published:** 2018-07-18

**Authors:** Monica Coll, Carles Ferrer-Costa, Sara Pich, Catarina Allegue, Emilio Rodrigo, Gema Fernández-Fresnedo, Paloma Barreda, Jesus Mates, Angel Luis Martinez de Francisco, Israel Ortega, Anna Iglesias, Oscar Campuzano, Eduardo Salas, Manuel Arias, Ramon Brugada

**Affiliations:** 1 Cardiovascular Genetics Center, IDIBGI, Dr. Trueta University Hospital, Parc Hospitalari Martí i Julià, Edifici M2, Salt, Spain; 2 Scientific Department, Gendiag.exe., Barcelona, Spain; 3 IFIMAV-Servicio de Nefrología del Hospital Universitario Marqués de Valdecilla, Santander, Spain; 4 Cardiology Service, Dr. Trueta University Hospital, Girona, Spain; Indiana University, UNITED STATES

## Abstract

**Background:**

Patients with end-stage renal disease have very high mortality. In individuals on hemodialysis, cardiovascular deaths account for ~50% of all deaths in this population, mostly due to arrhythmia. To determine the causes of these arrhythmic deaths is essential in order to adopt preventive strategies. The main objective of this study was to investigate whether, the presence of QTc interval alterations, from electrolyte abnormalities or presence of rare genetic variants, could have a relationship with sudden arrhythmogenic deaths in end-stage renal disease patients.

**Methods:**

We recorded the pre- and post-dialysis QTc interval in 111 patients undergoing hemodialysis. In 47 of them, we analyzed 24 SCD-related genes including the most prevalent genes associated with long QT syndrome using a custom resequencing panel.

**Results:**

We found a positive although not significant association between the presence of long QTc and mortality in a subset of end-stage renal disease patients. In addition, in five patients with long QTc only after dialysis (21.7%) we detected rare potentially pathogenic genetic variants. Three out of these five carriers subsequently died suddenly.

**Conclusions:**

Genetic background may be determinant in the risk of sudden cardiac death in these patients. We recommend evaluating the QTc interval before and after hemodialysis, and performing a genetic analysis of individuals with long QTc after hemodialysis.

## Introduction

Chronic kidney disease (CKD), defined as either kidney damage or glomerular filtration rate (GFR) below 60ml/min/1.73m^2^ for ≥ 3 months[1], is a very relevant health entity. CKD has a prevalence of 4.7–8.1% in people ages 60 and older and of 0.5%^,^ in the population between the ages of 20 and 39 years of age [2, 3]. People with CKD are at higher mortality risk compared with the general population. In fact, cardiovascular deaths (CD) account for ~50% of all deaths in CKD, particularly in individuals on hemodialysis (HD)[4]. Most cardiovascular deaths in this patient population involves arrhythmic mechanisms[5]. The risk factors predisposing to sudden cardiac death (SCD) include low concentration of potassium in the dialysate, diabetes mellitus, advanced age, and undergoing dialysis session after the weekend break[6].

In some studies have reported that HD sessions may induce altered ventricular repolarization with QTc interval prolongation, which could be a potential arrhythmic risk for these patients [7]. The QTc interval prolongation in CKD may be induced by electrolyte level alteration. Thus, in end-stage renal disease (ESRD), prolongation of the QTc interval has always been believed related to changes in serum potassium, magnesium, calcium and phosphate levels during HD[7, 8], and specially linked to the composition of the hemodialysis bath, since the longest QTc interval duration occurs with low-potassium and low calcium dialysate[9]. However, long QTc intervals could also be caused by a combination of electrolyte and genetic factors, as it is well known that genetic variants in genes encoding ion channels may be responsible for QTC alterations and long QT Syndrome (LQTS). However, investigation of a genetic background potentially predisposing to long QT in ESRD has yet to be performed.

Thus, in order to further understand the mechanisms leading to arrhythmogenicity in HD patients we aim at investigating the QTc interval in ESRD patients both before and after HD, and its relation with the presence of rare genetic variants associated with LQTS.

## Materials and methods

### Sample selection and clinical data

The patients included in this study were selected from a cohort of patients treated for CKD at the Nephrology Department of Marqués de Valdecilla University Hospital (Santander, Spain). We included all outpatients (N = 143) undergoing HD therapy between May, 2011 and July, 2012 at the Nephrology Service of the Marques de Valdecilla University Hospital. All patients had a GFR of <15ml/min/1.73m^2^. We excluded individuals who were taking drugs known to prolong the QT interval, and those who had a pacemaker, bundle branch block or atrial fibrillation. Thirty-two of 143 patients met the exclusion criteria (22.38%), so 111 patients were finally included in the study (77.62%). We recorded the following demographic and clinical parameters: age, gender, race, height, weight, time on HD, disease etiology, classical cardiovascular risk factors, and cardiovascular and coronary events. Patients were followed up until December, 2014.

This study was approved by the Institutional Human Research Review Board of the Hospital, was performed conform the declaration of Helsinki and all patients gave written informed consent to be included.

### Dialysis

We performed haemodiafiltration with High Flux Helixone (Fresenius Medical Care, Germany) for four hours per session, with a mean blood flow 350 ± 40 mL/min and mean weight loss of 2.1 ± 0.3 Kg. All patients were treated with a dialysis regimen of bicarbonate dialysis with the following electrolyte composition in the bath (mmol/L): 140 Na^+^, 1.5 K^+^, 1.25 Ca^2+^, 0.5 Mg^2+^. Drug therapy was allowed during hemodialysis, with the exception of isotonic saline infusion and sodium heparin.

### Electrocardiographic assessment

All enrolled patients underwent a resting, high-quality 12-lead ECG recording before and after the HD session in a standard setting (EGC recorder Cardiosoft model V6.0 with a CAM-14 module). The QT interval was measured by the investigators for each patient and was corrected for patient heart rate using Bazett’s formula: QTc = QT/√RR (ms), where QTc is the corrected value of the QT interval. ECG was recorded for a single HD session per patient. The recorded ECG was only used if the HD session took place without any unusual incidents and if the patient had been free of interfering factors (drugs affecting QT, fever, diarrhea, vomiting) for the previous 24 hours.

The patients were classified in four groups depending on the QTc value before and after HD. A QTc of >460ms was considered prolonged[10]. Group 1, NNQTc (Normal-Normal QTc), included patients without prolonged QTc before or after the HD; Group 2, NLQTc (Normal-Long QTc), included patients with normal QTc before and prolonged QTc after HD; Group 3, LNQTc (Long-Normal QTc), included patients with prolonged QTc before and normal after HD; and Group 4, LLQTc (Long-Long QTc), included patients with a long QTc both before and after HD.

### Laboratory analysis

A venous blood sample was drawn by phlebotomy immediately before and after HD sessions, collected in a sterile tube, placed immediately on ice, centrifuged on site within 90 min and frozen at -20°C. We quantified serum sodium, potassium, calcium, and magnesium ion concentrations using an ABL80 FLEX analyzer (Radiometer, Iberia). The following values were considered normal: Sodium, 135-145mEq/L; potassium, 3.5-5mEq/L; calcium, 8.1–10.4 mg/dL; magnesium, 1.6–2.4mg/dL.

### Genetic analysis

DNA samples were obtained from 38 out of the 41 patients with long QTc. In 3 patients the DNA quality precluded genetic analysis and we could not obtain a second DNA sample from them as they had died by the time we performed the genetic analysis. Moreover, we obtained DNA sample from 9 patients with no QTc prolongation.

#### DNA isolation

Genomic DNA was extracted from whole blood using the Chemagic MSM system I (Chemagic human blood). The concentration was checked using a Qubit 2.0 Fluorometer (Life Technologies) and purity was evaluated by measuring the 260/280 absorbance ratios (~ 1.8) using a NanoDrop1000 spectrophotometer (Thermo scientific). We used 1% agarose gel to confirm that that there was no DNA degradation.

#### Custom resequencing panel

The custom panel design included genes known to be involved in SCD-related pathologies (SudDinCode®, Ferrer in Code). The genomic coordinates of these genes were uploaded to the eArray web-based probe design tool (Agilent Technologies, Inc.). All isoforms described in the UCSC browser were included in the design. The biotinylated cRNA probe solution was manufactured by Agilent Technologies and provided as capture probes. The final size was 680 kbp of encoding regions and UTR boundaries. The coordinates of the sequence data is based on NCBI build 37 (UCSC hg19).

A 24-gene panel of genes associated with arrhythmogenic cardiac diseases was used: *ANK2*, *CACNA1C*, *CACNB2*, *CASQ2*, *CAV3*, *DES*, *DSC2*, *DSG2*, *DSP*, *GPD1L*, *HCN4*, *JUP*, *KCNE1*, *KCNE2*, *KCNH2*, *KCNJ2*, *KCNQ1*, *LMNA*, *PKP2*, *PRKAG2*, *RYR2*, *SCN4B*, *SCN5A* and *TGFB3*.

The technical characteristics of the NGS sequencing were: call rate 30X (99.8%), the remaining 0.2% of the genome was sequenced by conventional Sanger Sequencing. Sensibility and specificity (SNPs and small indels) were both 100%.

#### Bioinformatics analysis

Our secondary bioinformatics process includes a first-step that trims the FAST-Q files originated by the MiSeq platform using a method developed by Gendiag.exe. The trimmed reads are then mapped with GEM II. The output from the mapping step is combined and sorted, and uniquely and properly mapping read pairs are selected. Finally, the cleaned BAM file is annotated using SAMtools v.1.18, GATK v2.4, and an *ad hoc* method developed to generate the first raw VCF files. Variants are annotated with dbSNP IDs[11]; Exome Variant Server, NHLBI GO Exome Sequencing Project (ESP), Seattle, WA and the 1000 Genomes browser[12], in-home database IDs and Ensemble information, where available. Exome Aggregation Consortium (ExAC) was also consulted.

An algorithm developed by Gendiag.exe was used to determine the pathogenicity of the detected variants according to the following criteria: variants with minor allele frequency (MAF) <1% in the general population and the status of “probably damaging” in Provean and Mutation Taster2 prediction were considered as rare/uncommon variants. The potential pathogenicity of missense variants was evaluated *in silico* using Provean and Mutation Taster2[13]. The performance of Provean is comparable to popular tools such as SIFT or PolyPhen-2[14, 15]. As supplemental data we used a UniProt database to consult the level of conservation between different species [16]. An in-house database of variants was used as an internal control.

Rare genetic variants in genes related to long QT syndrome, and variants of uncertain significance (VUS) were confirmed by Sanger Sequencing.

### Statistical analysis

Continuous variables were expressed as the mean ± standard deviation, and categorical variables were expressed as percentages. Differences between two related samples were evaluated using a Paired-Samples T-test. We compared groups of continuous variables using ANOVA and of categorical variables using the χ^2^-test. A P-value of <0.05 was considered statistically significant.

Survival analysis was conducted using the Log Rang Test and Kaplan-Meyer by software R (version 2.12).

Statistical analyses were performed using SPSS Statistics for Windows, Version 19.0 (IBM Corp. Released 2010. IBM SPSS Statistics for Windows, Version 19.0. Armonk, NY: IBM Corp) and R (http://r-project.org).

## Results

### Hemodialysis-related changes in QTc and prognostic implications

We recruited a total of 111 patients undergoing regular hemodialysis, of which 70 had normal QTc (63.1%; NNQTc), 24 had long QTc only after dialysis (21.6%; NLQTc), 8 had long QTc only before dialysis (7.2%;, LNQTc) and 9 had long QTc both before and after dialysis (8.1%; LLQTc). The average age was 66.27 (63.44–69.1), and 81/111 (73%) were male. We observed no significant differences between the four groups in terms of age (p = 0.24), gender (p = 0.78), diabetes (p = 0.82) or hypertension (p = 0.24) (Table 1, Table 2 and S1 Table).

**Table 1:** Basic characteristics of the entire cohort and of the sequencing group.

		Overall	NNQTc	NLQTc	LNQTc	LLQTc	*P*-value
***Variable***	*unit*	**111 dialysis patients**					
**Sample size**	N (%)	111 (100%)	70 (63.1%)	24 (21.6%)	8 (7.2%)	9 (8.1%)	
**Diabetes**	N (%) *	32 (30.5%)	20 (29.4%)	8 (38.1%)	2 (28.6%)	2 (22.2%)	0.82
**Hypertension**	N (%) *	88 (83.8%)	58 (85.3%)	15 (71.4%)	6 (85.7%)	9 (100%)	0.24
**Age (years old)**	Mean (95%CI)	66.27 (63.44–69.1)	64.27 (60.38–68.17)	68.17 (62.41–73.93)	68.88 (61.6–78.14)	73.56 (66.89–80.22)	0.24
**Male Gender**	N (%)	81 (73%)	51 (72.90%)	17 (70.80%)	7 (87.50%)	6 (66.70%)	0.78
**QTc before HD** **(ms)**	Mean (95%CI)	426.05 (419.14–432.95)	409.44 (402.28–416.6)	435.5 (426.72–444.28)	481.38 (471.58–491.18)	480.78 (468.08–493.49)	
**QTc after HD** **(ms)**	Mean (95%CI)	440.66 (432.54–448.78)	416.74 (411.24–422.25)	493.54 (478.32–508.76)	434 (412.15–455.85)	491.56 (477.67–505.45)	
**Deaths**	N (%)	34 (30.63%)	14 (20%)	9 (37.5%)	5 (62.5%)	6 (66.6%)	0.008
**LVEF**	Mean (95%CI)	55.73 (53.62–57.83)	56.46 (53.83–59.09)	55.57 (50.49–60.64)	50 (31.9–68.1)	54 (48.26-58-74)	0.59

**Table 2:** Basic characteristics of the entire cohort and of the sequencing group.

		Overall	NNQTc	NLQTc	LNQTc	LLQTc	*P*-value
Variable	Unit	47 patients sequenced by NGS					
**Sample size**	N	47	9	23	6	9	
**Diabetes**	N (%) †	10 (27.02%)	1 (12.5%)	7 (35%)	0	2 (22.2%)	0.45
**Hypertension**	N (%) †	30 (73.17%)	7 (87.5%)	14 (70%)	4 (80%)	9 (100%)	0.14
**Age (years old)**	Mean (95%CI)	68.32 (64.67–71.97)	63.56 (53.99–73.12)	67.65 (61.73–73.58)	70.17 (58.07–82.27)	73.56 (66.89–80.22)	0.25
**Male Gender**	N (%)	30 (73.2%)	7 (77.8%)	17 (73.9%)	5 (83.3%)	6 (66.7%)	0.86
**QTc before HD (ms)**	Mean (95%CI)	440.49 (428.65–452.33)	386 (357.01–414.99)	436 (426.87–445.13)	479 (466.01–491.99)	480.78 (468.07–493.49)	
**QTc after HD (ms)**	Mean (95%CI)	470.57 (457.37–483.78)	414.89 (401.41–428.37)	494.52 (478.73–510.32)	430.84 (400.93–460.7)	491.56 (477.66–505.45)	

Ms, milliseconds; NNQTc, NORMAL-NORMAL QTc; NLQTc, NORMAL-LONG QTc; LNQTc, LONG-NORMAL QTc; LLQTc, LONG-LONG QTc.

* Data available for 68, 21, 7 and 9 patients from the NNQTc, NLQTc, LNQTc and LLQTc groups, respectively.

† Data available for 8, 20, 5 and patients from the NNQTc, NLQTc, LNQTc and LLQTc groups, respectively.

The potassium and magnesium levels decreased significantly following HD in the four groups (Table 3 and Table 4). Potassium levels were within normal range in pre-dialysis (except for LLQTc group) and decreased to a value below the normal range in post-dialysis. Magnesium levels were above the normal range in pre-dialysis and decreased to the normal range in post-dialysis. Calcium levels increased significantly after HD in all groups except NLQTc group, although the values were within normal range in all groups. Sodium levels after HD were not significantly different to those before HD in any group, and were also within the normal rang.

**Table 3:** Electrolyte levels of the entire cohort.

111 dialysis patients												
Variable	NNQTc (N = 70)			NLQTc (N = 24)			LNQTc (N = 8)			LLQTc (N = 9)		
	Pre-HD	Post-HD	*P*-Value	Pre-HD	Post-HD	*P*-Value	Pre-HD	Post-HD	*P*-Value	Pre-HD	Post-HD	*P*-Value
**Sodium**	138.97 (138.26–139.68)	139.01 (138.62-139-4)	0.858	139.13 (137.92–140.33)	139.25 (138.21–140.29)	0.845	137.5 (134.60-140-40)	136.86 (135.4–138.31)	0.744	137.89 (135.63–140.14)	138.22 (136.74–139.7)	0.74
**Potassium**	4.96 (4.75–5.17)	3.12 (3.03–3.22)	P < .001	4.9 (4.56–5.24)	3.06 (2.92–3.2)	P < .001	4.48 (3.62–5.33)	2.97 (2.67–3.28)	P < .001	5.18 (4.43–5.93)	3.16 (2.86–3.46)	P < .001
**Calcium**	8.65 (8.51–8.78)	8.93 (8.8–9.06)	P < .001	8.62 (8.27–8.98)	8.89 (8.71–9.07)	0.109	8.4 (7.63–9.17)	9.23 (8.4–10.06)	0.012*	8.36 (7.83–8.88)	8.94 (8.49–9.4)	0.002*
**Magnesium**	2.51 (2.4–2.62)	2.13 (2.08–2.17)	P < .001	2.43 (2.28–2.58)	2.13 (2.05–2.2)	P < .001	2.35 (2.09–2.61)	2.07 (1.89–2.25)	0.002	2.4 (1.91–2.89)	2.02 (1.8–2.24)	0.022*

**Table 4:** Electrolyte levels of the sequenced groups.

Variable	NNQTc (N = 9)			NLQTc (N = 23Ɨ)			LNQTc (N = 6 ƗƗ)			LLQTc (N = 9)		
	Pre-HD	Post-HD	*P-*Value	Pre-HD	Post-HD	*P*-Value	Pre-HD	Post-HD	*P-*Value	Pre-HD	Post-HD	*P-*Value
**Sodium**	140.11 (137.96–142.27)	140.11 (138.81–141.41)	1	139.09 (137.82–140.35)	139.3 (138.23–140.38)	0.742	139 (136.8–141.2)	137 (134.68–139.32)	0.047	137.89 (135.63–140.14)	138.22 (136.74–139.7)	0.74
**Potassium**	4.83 (4.18–5.48)	3.03 (2.78–3.29)	P < .001	4.87 (4.52–5.22)	3.05 (2.91–3.2)	P < .001	4.57 (3.31–5.82)	3.04 (2.58–3.5)	0.003	5.18 (4.43–5.93)	3.16 (2.86–3.46)	P < .001
**Calcium**	8.61 (8.23–8.99)	8.91 (8.62–9.2)	0.097	8.6 (8.23–8.96)	8.89 (8.7–9.07)	0.098	8.35 (7.21–9.49)	8.9 (7.8–9.8)	0.065	8.36 (7.83–8.88)	8.94 (8.49–9.4)	0.002*
**Magnesium**	2.83 (2.45–3.22)	2.22 (2.12–2.33)	0.003*	2.41 (2.26–2.57)	2.13 (2.05–2.2)	P < .001	2.37 (2–2.73)	2.12 (1.85–2.39)	0.003	2.4 (1.91–2.89)	2.02 (1.81–2.23)	0.022*

Ɨ 22 available patients in potassium and magnesium levels.

ƗƗ 5 available patients in all the electrolyte levels. Sodium and potassium levels are expressed in mEq/L and calcium and magnesium levels are expressed in mg/gL. NNQTc, NORMAL-NORMAL QTc; NLQTc, NORMAL-LONG QTc; LNQTc, LONG-NORMAL QTc; LLQTc, LONG-LONG QTc. Values expressed as Mean (95%-CI).

*P value<0.05.

We found no significant differences in electrolyte levels between the four groups, except for post-dialysis sodium (p = 0.017) (S2 Table).

**Fig 1:**
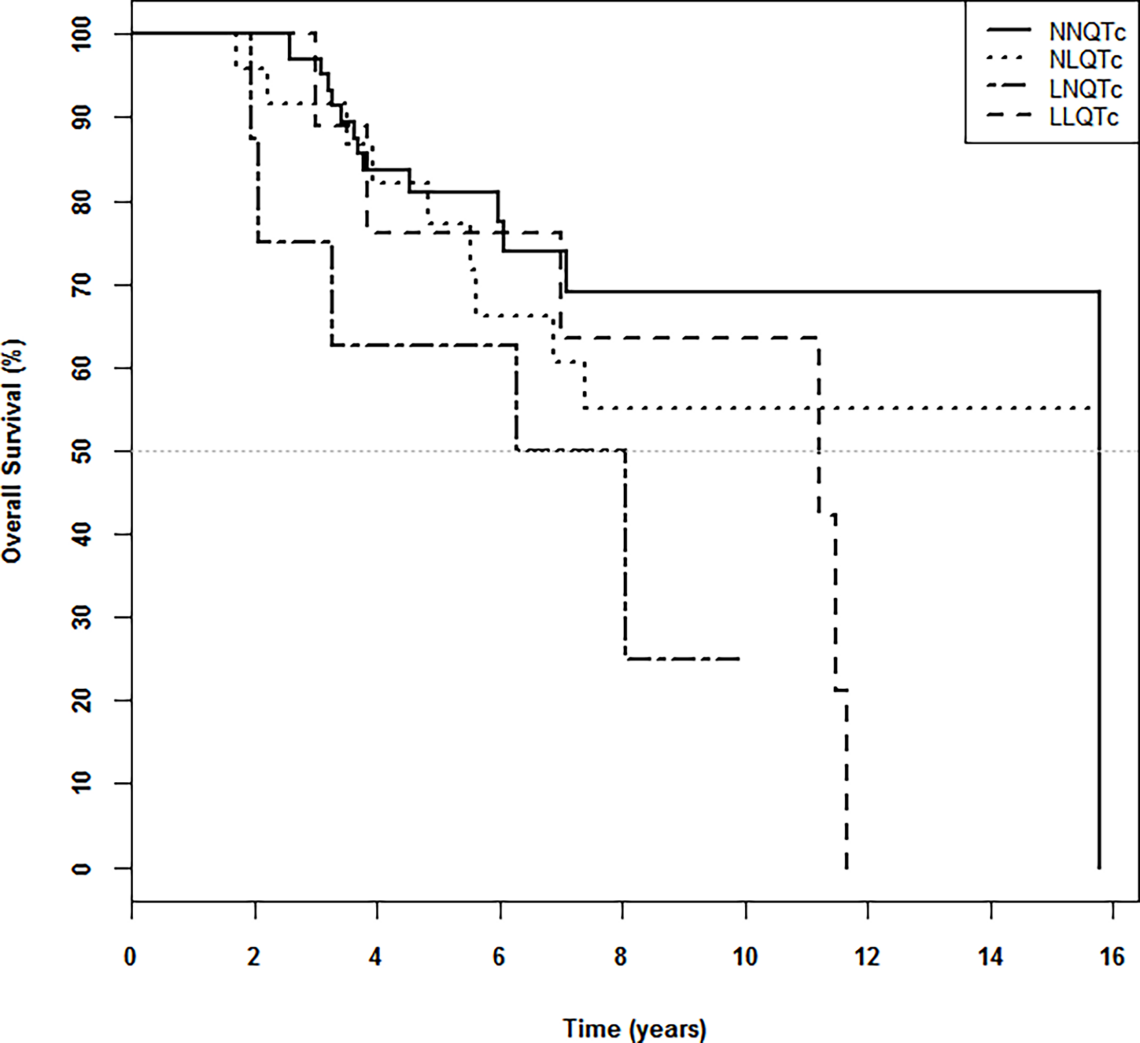
Kaplan-Meier curves for the 4 groups. NNQTc: QTc interval normal pre-HD and post-HD; NLQTc: QTc interval normal pre-HD and long post-HD; LNQTc: QTc interval long pre-HD and normal post-HD; LLQTc: QTc interval long pre-HD and post-HD.

Mortality: Overall, 30.63% of patients died during the study, with patients with long QTc having higher mortality than the control group (Table 1, Table 2 and S3 Table). The highest mortality was observed in group 4 (LLQTc; 66.66%) followed by group 3 (LNQTc; 62.5%) and group 2 (NLQTc; 37.5%). The lowest mortality was observed in patients in group 1 (NNQTc; 20%). QTc was significantly longer, both pre- and post- HD, among patients who died during follow-up than among those who survived (S4 Table). After Log Rank analysis (Table 5) no significant differences were identified (p = 0.144) however focusing on the Kaplan-Meyer curve the NNLQTc group showed higher survival than the groups with long QTc pre or post-HD. Additionally the groups of NLQTc and LNQTc showed the lower survival (Fig 1).

**Table 5:** Kaplan-Meier survival analysis.

Group	N	1 year	3 years	5 years	10 years	P-value
NNQTc	70	100%	95,1%±2,8%	77,5%±6,3%	69%±8%	0,144
NLQTc	24	95,8%±4,1%	86,8%±7,1%	71,7%±9,9%	55,1%±11,3%	
LNQTc	8	87,5%±11,7%	62,5%±17,1%	50%±17,7%	25%±19,8%	
LLQTc	9	88.9%±10,57%	76,2%±14,8%	63,5%±16,9%	42,3%±20,6%	

NNQTc: QTc interval normal pre-HD and post-HD; NLQTc: QTc interval normal pre-HD and long post-HD; LNQTc: QTc interval long pre-HD and normal post-HD; LLQTc: QTc interval long pre-HD and post-HD. N: number of patients included in the corresponding group. P-value obtained from a Chi-squared test.

### Relationship between rare genetic variants in long QT syndrome genes and changes in QTc induced by hemodialysis

We sequenced a total of 47 patients, as follows: 9 patients from NNQTc group, 23 patients from NLQTc, 6 patients from LNQTc group, and 9 patients from LLQTc group. It was not possible to sequence one patient from NLQTc group, and two patients from LNQTc group due to poor DNA quality and the inhability of obtaining a new DNA sample (they died before the genetic testing was performed) (Table 1 and Table 2).

#### Potentially pathogenic variants

**Fig 2:**
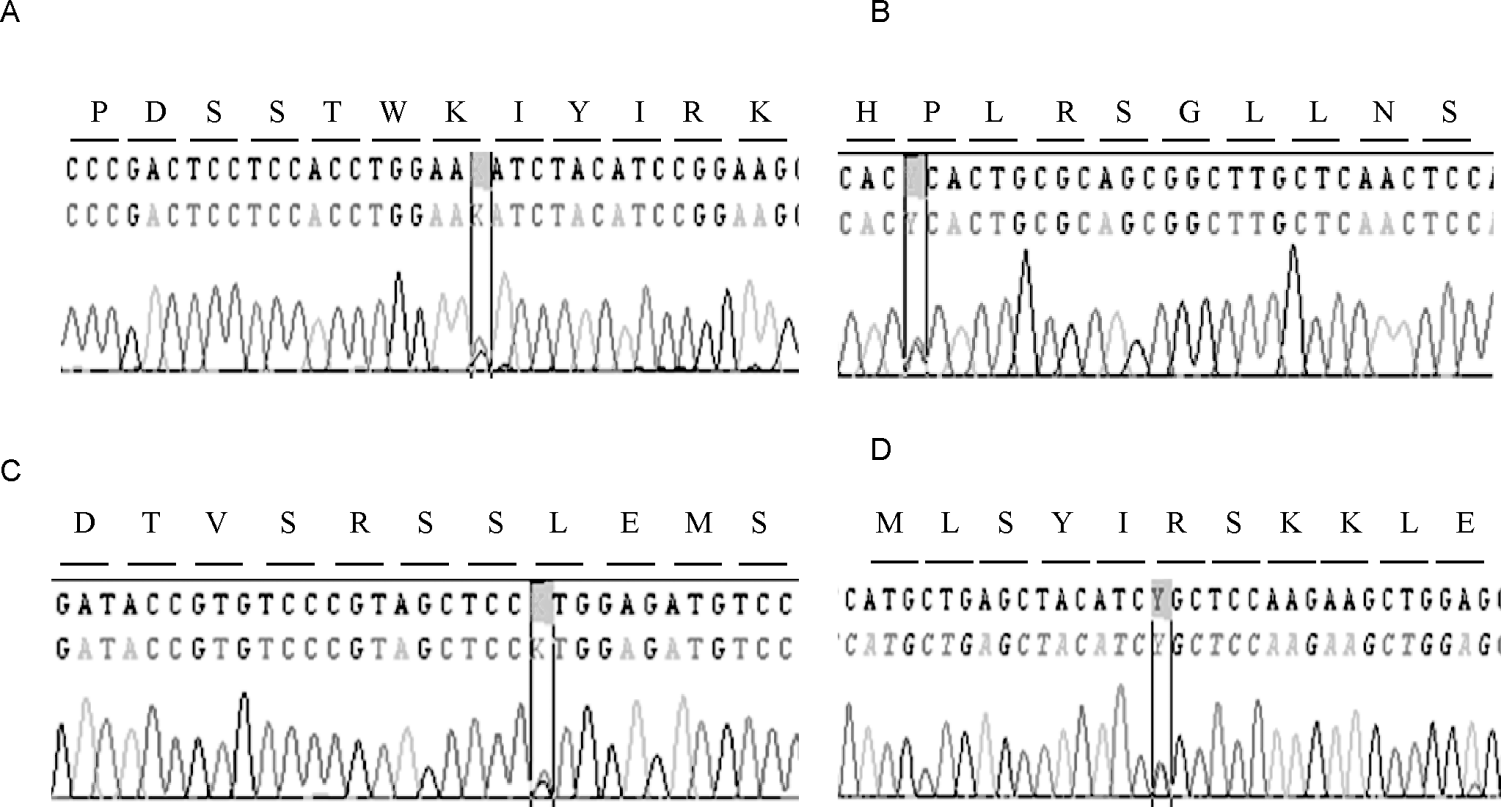
Forward and reverse electropherograms of the uncommon variants identified. A: *KCNQ1*_p.K393N; B: *KCNH2*_p.P310S; C: *SCN5A*_p.L461V; D: *KCNE1*_p.R67C.

Ultra-sequencing genetic analysis identified five rare variants in genes related with long QT syndrome in five patients from the NLQTc group (Fig 2, Table 6 and Table 7). Index case 83 carried a private variant (*KCNH2*_p.P310S) not previously reported or identified in any of the available variant databases. Two unrelated patients (index cases 76 and 84) carried a variant (*KCNQ1*_p.K393N) that had previously been reported to be associated with long QT syndrome (CM078293), although via a different nucleotide change (cytosine, not a thymine). We identified a variant in the *SCN5A* gene (*SNC5A*_p.L461V) in case 86, previously associated with sudden adult death syndrome[17, 18]. Finally, we detected a variant (*KCNE1*_p.R67C) in index case 94 that had previously been reported as being associated with long QT syndrome [17].

**Table 6:** Description of the five uncommon variants identified, plus available clinical data. Exome Sequencing Project and ExAC.

Index Case	Gene	Isoform	SNP	HGMD	Protein change	cDNA change	MAF (%) EA/All	ExAC (%)	Disease	PolyPhen2	Condel
**76**	*KCNQ1*	NM_000218.2	rs12720457	-	p.Lys393Asn	c.1179G>T	0.0814/0.0692	0.1098	LQTS	Possibly damaging (0.770)	Neutral (0.462)
**83**	*KCNH2*	NM_000238.3	-	-	p.Pro310Ser	c.928C>T	-	-	-	Benign (0.112)	Neutral (0.018)
**84**	*KCNQ1*	NM_000218.2	rs12720457	-	p.Lys393Asn	c.1179G>T	0.0814/0.0692	0.1098	LQTS	Possibly damaging (0.770)	Neutral (0.462)
**86**	*SCN5A*	NM_198056.2	rs41313697	CM061953	p.Leu461Val	c.1381T>G	0.0241/0.294	0.1163	SADS	Benign (0.08)	Neutral (0.392)
**94**	*KCNE1*	NM_000219.4	-	CM097703	p.Arg67Cys	c.199C>T	-	2.473.10–3	LQTS	Probably damaging (1)	Deleterious (1)

**Table 7:** Description of the five uncommon variants identified, plus available clinical data. Exome Sequencing Project and ExAC.

Index Case	Gender	Age	QTc pre/post	Diabetes	Hypertension	Status	Cause of death	General etiology	Specific etiology
**76**	Male	74	440/626	No	Yes	Death	Unknown	Vascular	Nephroangiosclerosis and/or ischemic nephropathy
**83**	Male	67	453/474	Yes	No	Death	Cardiac arrest	Vascular	Nephroangiosclerosis
**84**	Male	43	420/480	Yes	Yes	HD	-	Systemic	Nephropathy diabetic
**86**	Female	85	429/469	Yes	Yes	HD	-	Vascular	Nephroangiosclerosis and nephropathy diabetic
**94**	Female	80	460/461	No	Yes	Death	Cardiac arrest	Vascular	Nephroangiosclerosis

Abbreviations: Age is expressed in years. MAF: minor allele frequency; EA: European American; SNP: single nucleotide polymorphism. ExAC: Exome Aggregation Consortium, Cambridge, MA (URL: http://exac.broadinstitute.org) [(March, 2017) accessed]. SADS: Sudden adult death syndrome.

#### Variants of uncertain significance

Analysis of the 24 genes detected 20 common and uncommon variants in 16 patients (34.04%) (S5 Table), 11 of which (69%) were indels and 5 (31%) were single-point variants. We observed variants of uncertain significance in 7 patients with NNQTc, 6 with NLQTc, 3 with LNQTc and 4 with LLQTc. We identified uncommon variants in *DSP*, *JUP*, *CACNB2*, *RYR2* and *CACNA1C* (2 cases) genes. Two cases with uncommon variants (index cases 10 and 102) carried the common rs17133512 variant in *DSP*. We were unable to identify any uncommon variants in 31 cases.

## Discussion

For the past four decades, patients who are on renal replacement therapy have been known to have significant cardiovascular risk[19]. Most studies that have explored the basis of this risk have focused on fluctuations in blood ion levels as a result of HD, and their relation with increased QTc following HD. We have observed similar post-HD variations in ion levels as previously published[20]. However, the correlations of these ion levels with QTc are a matter of controversy. While Howse *et al*.[21], did not identify a correlation, Alabd *et al*.[9] reported a correlation between post-dialysis QTc and a decrease in serum levels of potassium and magnesium after dialysis.

In order to shed some light in this issue, we have investigated the QTc duration before and after HD and included genetic analysis in patients with prolonged QTc interval. We identified twenty-four patients (21.6%) who had a prolonged QTc interval only after the HD session. This percentage increased to 29.7% when we added those patients with long QTc before and after HD (groups NLQTc and LLQTc together), and to 36.9% when we added those patients with long QTc before HD (LNQTc, NLQTc and LLQTc). These data are very similar to those published by Genovesi *et al*. in a cohort of 122 patients, in which they reported an incidence of QTc prolongation of 36% in patients on HD, as measured before, during and after dialysis[22]. Similar to previous studies, the length of the QTc interval was not associated with gender, age or hypertension[20]. In contrast to other reports, the QTc interval in our cohort was not associated with the presence of diabetes [23].

We identified a clear correlation between the presence of a long QTc interval and mortality in patients undergoing HD. To our knowledge, this is the first report of an association between QTc interval and mortality in HD patients. Accordingly, those patients who died during the study showed a higher QTc both pre- and post-HD than the surviving patients, which indicates that a higher QTc value is an indicator or a bad prognosis in HD patients.

**Fig 3:**
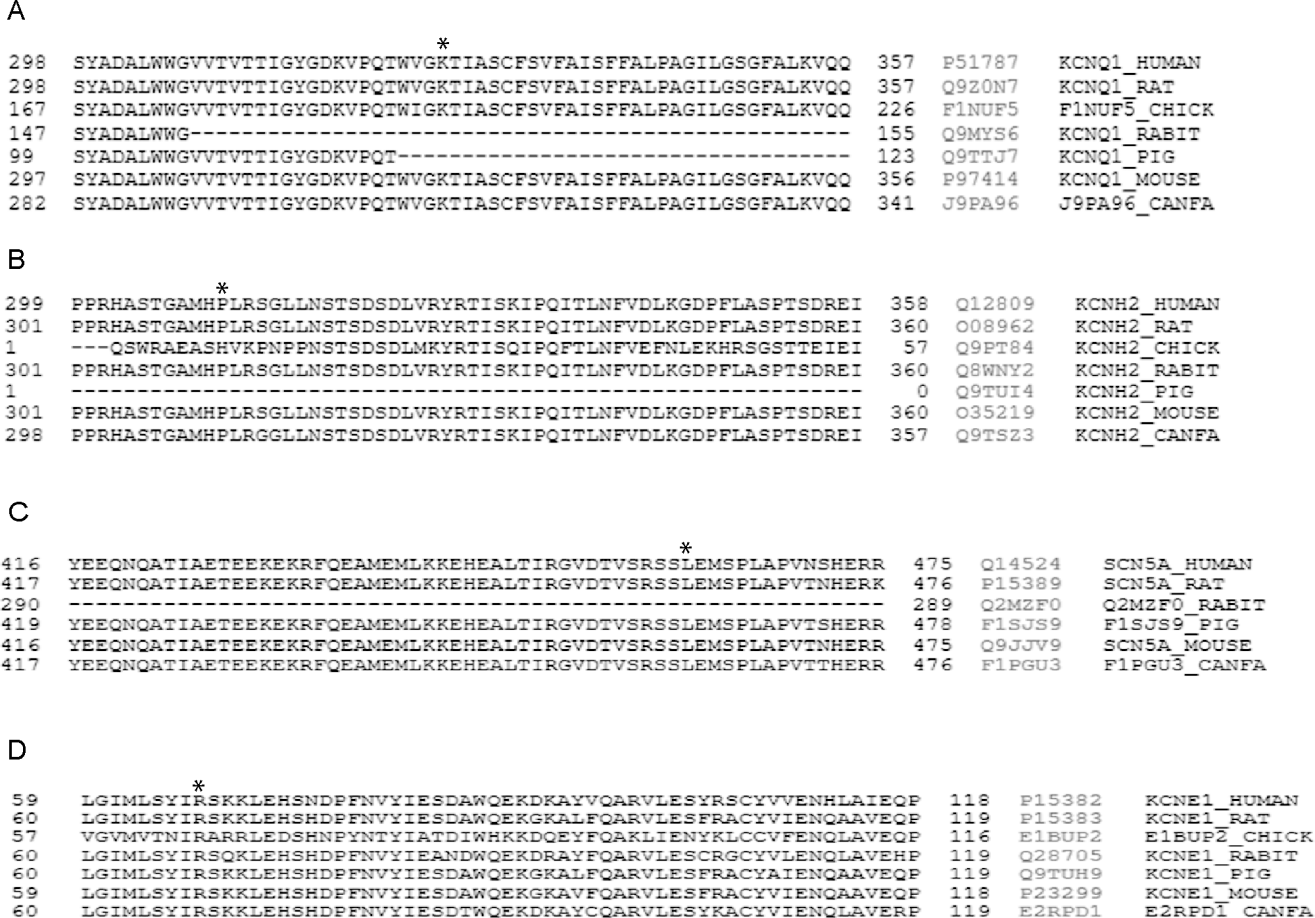
Sequence alignment for each variant in different species. The position of the first amino acid of each sequence is indicated on the left, and the Uniprot reference for each protein is shown on the right. A: *KCNQ1*_p.K393N; B: *KCNH2*_p.P310S; C: *SCN5A*_p.L461V; D: *KCNE1*_p.R67C.

Genetic analysis of the main LQTS genes enabled the identification of five potentially pathogenic variants, which may be associated with prolongation of the QTc interval, in patients with prolonged QTc interval only after HD (NLQTc group). While we were unable to perform segregation studies, the high level of conservation between species indicates that these nucleotide changes may have an important role in protein function (Fig 3). Supporting this potential pathogenic role, *in silico* analysis also predicted a deleterious effect.

In addition, we identified a large number of variants of unknown significance (20 variants, observed in 16 of 47 cases), of which 6 were uncommon. The role of variation in these genes in patients with ESRD merits further studies.

The considerably higher frequency of variants in sodium and potassium channels in this patient group compared to the general population is intriguing. Gene expression databases indicate that potassium channels (*KCNQ1*, *KCNH2* and *KCNE1* genes) are expressed in renal cells (GTEX and the human protein atlas database). The function of basolateral Kv7.1 (*KCNQ1)* within the various segments of the nephron is still to be determined. However, these channels could certainly be responsible for recycling K^+^ across the basolateral membrane, and for maintaining an ion potential gradient, which serves to drive Na^+^ secretion in the nephron[24]. Additionally, it seems likely that Kv7.1 plays a role in K^+^ reabsorption, in the context of a low-K^+^ diet for example[25]. The pore-forming partner(s) of *KCNE1* and the role of the respective channel complexes in the kidney remain unknown [26]. Finally, *KCNH2* is also expressed in the kidney, although the function of its product, Kv11.1, has not yet been studied in this organ. This novel mutation in *KCNH2* would be a good candidate variant to test function in-vitro studies to elucidate their possible pathogenic role.

## Conclusions

In conclusion, we provided evidence that the presence of long QTc interval in patients, who are undergoing dialysis, may be an indicator of risk for SCD. We did not observe a relationship between changes in electrolyte levels and the increment in QTc observed in some patients following HD. In fact, despite using an ion-balanced hemodialysis bath, we observed similar changes in electrolytes following HD to those previously reported. This question the relevance of these electrolytes changes for prolongation of the QT interval in HD.

The presence of a prolonged QT interval was associated with the identification of pathogenic genetic variants in ion channels, especially in those ESRD patients who presented with a prolonged QT interval only after HD (NLQTc group). In addition, 3 out of 5 individuals in this group died suddenly. This raises the hypothesis that there may be a genetic predisposition to sudden cardiac death which may be identified by electrocardiographic and/or genetic investigation.

## Supporting information

S1 Table Clinical data for all 111 studied cases. (DOCX)

S2 Table Electrolyte levels of the entire cohort and the sequenced groups. *P value<0.05.
(DOCX)

S3 Table Classification and duration in the study of the 111 studied cases. (DOCX)

S4 Table QTc values and electrolyte levels of the entire cohort for alive and deceased patients at the end of the study. (DOCX)

S5 Table Variants of uncertain significance. (DOCX)
