# Supplementary material for: Role of genetic and electrolyte abnormalities in prolonged QTc interval and sudden cardiac death in end-stage renal disease patients
Source: PLoS One. 2018 Jul 18;13(7):e0200756. doi: 10.1371/journal.pone.0200756 (PMC6051653; doi:10.1371/journal.pone.0200756)
Supplement: S1 Table — (DOCX) [file pone.0200756.s001.docx]

**SUPPLEMENTAL MATERIAL**

**S1 Table.** Clinical data for all 111 studied cases

| **Group** | **Index Case** | **QTc pre** | **QTc post** | **Na+ Pre** | **K^+^ Pre** | **Ca^2+^ Pre** | **Mg^2+^ Pre** | **Na^+^ Post** | **K^+^ Post** | **Ca^2+^ Post** | **Mg^2+^ Post** | **Age** | **Gender** | **Diabetes** | **Hypertension** | **Status** | **Etiology** | **NGS** |
| --- | --- | --- | --- | --- | --- | --- | --- | --- | --- | --- | --- | --- | --- | --- | --- | --- | --- | --- |
| 1 | 1 | 408 | 417 | 138 | 4,1 | 9,1 | 2,6 | 138 | 3 | 9,2 | 2,3 | 68 | M | + | + | 1 | 1 | - |
| 1 | 2 | 391 | 452 | 137 | 5,3 | 8,2 |  | 138 | 3,9 | 8,4 | 2,4 | 81 | M | - | + | 2 | 2 | - |
| 1 | 3 | 437 | 426 | 138 | 5,4 | 8,3 | 2,6 | 140 | 3,1 | 8,9 | 2,4 | 57 | F | - | + | 1 | 3 | - |
| 1 | 4 | 413 | 426 | 139 | 4,9 | 8,8 | 2,9 | 139 | 2,8 | 9,4 | 2,3 | 75 | M | - | + | 1 | 3 | + |
| 1 | 5 | 375 | 402 | 142 | 4,4 | 8,9 | 2,5 | 140 | 3 | 8,6 | 2,2 | 76 | F | - | - | 2 | 4 | - |
| 1 | 6 | 417 | 392 | 140 | 4,7 | 8,4 | 1,9 | 139 | 3 | 8,4 | 1,9 | 68 | M | - | + | 2 | 5 | - |
| 1 | 7 | 393 | 393 | 143 | 6,1 | 8,4 | 2,3 | 140 | 2,9 | 8,9 | 2 | 81 | M | - | + | 1 | 2 | - |
| 1 | 8 | 442 | 375 | 138 | 7 | 9,2 | 2,6 | 138 | 3,7 | 9,1 | 2,3 | 77 | M | - | + | 2 | 2 | - |
| 1 | 9 | 400 | 437 | 137 | 5,7 | 8,5 | 3 | 140 | 3 | 8,7 | 2,1 | 74 | M | - | + | 1 | 2 | + |
| 1 | 10 | 384 | 392 | 139 | 5,5 | 9 | 2,8 | 143 | 2,8 | 9,3 | 2,3 | 54 | M | - | + | 1 | 3 | + |
| 1 | 11 | 384 | 408 | 135 | 3,8 | 7,9 | 3,3 | 138 | 2,6 | 8,2 | 2,2 | 76 | M | - | - | 1 | 5 | + |
| 1 | 12 | 413 | 424 | 142 | 5,9 | 8 | 2 | 141 | 3,4 | 9,1 | 2,2 | 57 | M | - | + | 1 | 3 | + |
| 1 | 13 | 388 | 417 | 138 | 4,5 | 8,1 | 2 | 139 | 2,9 | 8,5 | 2 | 79 | M | - | + | 1 | 3 | - |
| 1 | 14 | 413 | 437 | 142 | 4,3 | 8,8 | 2,2 | 140 | 3,5 | 9,2 | 2 | 44 | F |  |  | 3 | 5 | + |
| 1 | 15 | 393 | 393 | 139 | 5,3 | 9,1 | 2,6 | 139 | 3,1 | 9,4 | 2,2 | 73 | M | - | + | 1 | 3 | - |
| 1 | 16 | 447 | 436 | 142 | 5,4 | 8,6 | 3,1 | 139 | 3 | 9,2 | 2,5 | 33 | M | - | + | 3 | 3 | - |
| 1 | 17 | 460 | 453 | 144 | 3,9 | 8,8 | 2,1 | 140 | 2,8 | 8,6 | 2 | 85 | M | - | + | 1 | 3 | - |
| 1 | 18 | 384 | 402 | 143 | 5 | 8,1 | 2,2 | 141 | 2,8 | 9,1 | 2,2 | 48 | M | - | + | 3 | 3 | - |
| 1 | 19 | 393 | 402 | 136 | 5 | 9,1 | 3 | 138 | 3,3 | 9,2 | 2,3 | 79 | M | + | + | 1 | 2 | - |
| 1 | 20 | 384 | 400 | 142 | 4,7 | 8,7 | 2,6 | 141 | 3 | 8,7 | 2,1 | 81 | M | - | + | 1 | 5 | - |
| 1 | 21 | 402 | 447 | 139 | 3,7 | 8,6 | 2,2 | 142 | 2,8 | 8,4 | 2,1 | 71 | M | + | + | 1 | 1 | - |
| 1 | 22 | 367 | 393 | 142 | 4,8 | 8,3 | 2,2 | 141 | 3 | 8,4 | 2 | 79 | F | + | + | 1 | 1 | - |
| 1 | 23 | 459 | 392 | 143 | 3,4 | 8,5 | 2 | 140 | 3 | 8,5 | 2 | 78 | M | - | + | 1 | 3 | - |
| 1 | 24 | 431 | 436 | 134 | 5,7 | 8,5 | 3,1 | 140 | 3,3 | 8,6 | 2,2 | 77 | M | + | - | 2 | 1 | - |
| 1 | 25 | 450 | 450 | 141 | 3,5 | 9 | 2,4 | 139 | 2,9 | 8,8 | 2 | 79 | M | - | + | 2 | 3 | - |
| 1 | 26 | 415 | 408 | 142 | 6,2 | 7,6 | 2,4 | 137 | 3,4 | 9 | 2,3 | 28 | M | - | + | 1 | 1 | - |
| 1 | 27 | 393 | 413 | 139 | 4,3 | 8,7 | 2,4 | 139 | 3,5 | 9,4 | 2,2 | 77 | M | + | + | 1 | 1 | - |
| 1 | 28 | 398 | 426 | 140 | 4,3 | 8,8 | 2,8 | 140 | 3,1 | 8,9 | 2,3 | 74 | F | + | + | 1 | 1 | - |
| 1 | 29 | 426 | 431 | 140 | 4,7 | 9,1 | 2,5 | 139 | 2,9 | 9,1 | 2,1 | 76 | M | - | + | 1 | 3 | - |
| 1 | 30 | 412 | 413 | 141 | 5 | 9 | 2,6 | 141 | 2,7 | 10,3 | 2,2 | 22 | M | - | - | 3 | 4 | - |
| 1 | 31 | 380 | 413 | 140 | 5,8 | 9,3 | 2 | 140 | 3,3 | 10 | 2,1 | 63 | M | - | - | 2 | 6 | - |
| 1 | 32 | 436 | 417 | 139 | 3,7 | 8,7 | 3,1 | 143 | 3,2 | 9,1 | 2,4 | 73 | M | + | + | 1 | 1 | - |
| 1 | 33 | 459 | 424 | 138 | 4,3 | 8,8 | 3 | 139 | 2,8 | 9,7 | 2,3 | 59 | F | - | + | 3 | 3 | - |
| 1 | 34 | 408 | 389 | 140 | 4,3 | 8,5 | 2,5 | 138 | 3,2 | 8,4 | 2,1 | 82 | M | - | - | 1 | 5 | - |
| 1 | 35 | 450 | 459 | 141 | 5,4 | 8,9 | 2,2 | 141 | 3,7 | 9,4 | 2,1 | 75 | M | - | + | 2 | 2 | - |
| 1 | 36 | 386 | 434 | 139 | 3,3 | 8,2 | 2,3 | 139 | 2,7 | 8,4 | 2,1 | 79 | M | - | + | 1 | 2 | - |
| 1 | 37 | 424 | 447 | 142 | 5,6 | 8,6 | 3,1 | 139 | 3,5 | 8,6 | 2,2 | 72 | F | + | + | 1 | 2 | - |
| 1 | 38 | 409 | 402 | 140 | 5,8 | 9 | 2,4 | 136 | 3,2 | 9 | 2,1 | 68 | F | + | + | 2 | 1 | - |
| 1 | 39 | 338 | 400 | 141 | 5,1 | 8,7 | 2,7 | 137 | 3 | 9,4 | 2,2 | 38 | M | - | - | 3 | 1 | - |
| 1 | 40 | 400 | 344 | 138 | 6,4 | 8,4 | 3 | 139 | 3,1 | 9,1 | 2,2 | 75 | F | - | + | 1 | 1 | - |
| 1 | 41 | 424 | 442 | 142 | 4,5 | 9,1 | 3 | 139 | 2,9 | 9,4 | 2,2 | 25 | M | - | + | 3 | 2 | - |
| 1 | 42 | 402 | 431 | 141 | 3,9 | 8,8 | 2,3 | 139 | 3 | 9,3 | 2,3 | 58 | M | + | + | 1 | 1 | - |
| 1 | 43 | 379 | 393 | 143 | 5,5 | 8,2 | 3,5 | 142 | 3,3 | 8,8 | 2,4 | 61 | M | + | + | 1 | 1 | + |
| 1 | 44 | 396 | 400 | 143 | 4 | 9,4 | 2,6 | 138 | 2,7 | 8,9 | 2,1 | 53 | M | - | + | 1 | 7 | + |
| 1 | 45 | 413 | 398 | 138 | 5 | 8,4 |  | 139 | 3,8 | 9 |  | 37 | M | - | + | 1 | 3 | - |
| 1 | 46 | 393 | 393 | 136 | 4,8 | 8,7 | 2,1 | 138 | 3,4 | 8,4 | 2,1 | 38 | F | + | + | 1 | 2 | - |
| 1 | 47 | 426 | 437 | 138 | 5,8 | 9 | 3 | 139 | 3,3 | 9,3 | 2,5 | 69 | M | + | + | 1 | 1 | - |
| 1 | 48 | 450 | 437 | 138 | 4 | 8,8 | 2,2 | 138 | 3 | 8 | 1,9 | 40 | M | - | - | 1 | 1 | - |
| 1 | 49 | 402 | 393 | 141 | 4,7 | 9,2 | 2,6 | 141 | 2,8 | 9 | 2,2 | 42 | M | - | + | 3 | 4 | - |
| 1 | 50 | 450 | 450 | 129 | 5,3 | 8,4 | 2,1 | 139 | 3,2 | 9 | 2,1 | 59 | M | + | + | 1 | 1 | - |
| 1 | 51 | 409 | 423 | 138 | 4,3 | 8,9 | 1,9 | 139 | 2,9 | 8,6 | 1,9 | 81 | M | - | + | 1 | 2 | - |
| 1 | 52 | 400 | 413 | 137 | 4,9 | 9,7 | 2,9 | 139 | 3 | 8,2 | 2 | 53 | F | + | + | 1 | 1 | - |
| 1 | 53 | 436 | 378 | 140 | 5,7 | 9,2 | 2,2 | 140 | 4,2 | 9 | 2 | 58 | M | + | + | 1 | 1 | - |
| 1 | 54 | 292 | 417 | 141 | 3,9 | 8,9 | 3,2 | 140 | 3,2 | 8,6 | 2,4 | 78 | F | - | + | 1 | 5 | + |
| 1 | 55 | 400 | 413 | 141 | 3,7 | 7,6 | 1,8 | 139 | 2,9 | 7,6 | 1,7 | 55 | M | - | + | 1 | 3 | - |
| 1 | 56 | 359 | 445 | 135 | 5,1 | 9,7 | 2,2 | 138 | 2,5 | 9,2 | 1,8 | 65 | F | - | + | 2 | 3 | - |
| 1 | 57 | 442 | 440 | 138 | 5,7 | 7,3 | 2,4 | 136 | 2,8 | 8,4 | 2 | 44 | F | - | + | 1 | 5 | - |
| 1 | 58 | 413 | 413 | 132 | 6,7 | 7,5 | 1,8 | 137 | 4 | 8,5 | 1,8 | 46 | M | - | + | 3 | 4 | - |
| 1 | 59 | 439 | 439 | 135 | 5,1 | 9,2 | 1,8 | 138 | 2,3 | 9,1 | 1,8 | 45 | M | - | - | 1 | 4 | - |
| 1 | 60 | 449 | 422 | 142 | 5,4 | 7,2 | 2,4 | 139 | 2,9 | 8,2 | 1,9 | 66 | F | - | + | 1 | 3 | - |
| 1 | 61 | 425 | 428 | 137 | 5,3 | 8,6 | 2,8 | 137 | 3,3 | 9,7 | 2 | 79 | F | + | + | 2 | 1 | - |
| 1 | 62 | 382 | 418 | 136 | 5,9 | 8,5 | 3,3 | 136 | 3,3 | 10 | 2,2 | 69 | F | + | + | 1 | 1 | - |
| 1 | 63 | 432 | 380 | 134 | 6,9 | 6,9 | 2,5 | 138 | 3,6 | 7,9 | 2 | 74 | M | - | + | 2 | 2 | - |
| 1 | 64 | 400 | 372 | 135 | 6 | 9,1 | 2,3 | 137 | 3,6 | 9,9 |  | 84 | M | - | + | 1 | 2 | - |
| 1 | 65 | 413 | 425 | 137 | 5,8 | 8,5 | 2,2 | 135 | 4,2 | 8,8 | 2 | 81 | F | + | + | 2 | 1 | - |
| 1 | 66 | 414 | 441 | 140 | 3,2 | 9,4 | 2,2 | 141 | 2,5 | 8,9 | 1,9 | 44 | F | - | + | 1 | 1 | - |
| 1 | 67 | 382 | 409 | 132 | 3,5 | 9,7 | 2 |  |  | 10,4 | 2,3 | 57 | M | - | + | 3 | 2 | - |
| 1 | 68 | 451 | 442 | 140 | 5,5 | 8,6 | 2,2 | 137 | 2,6 | 8,6 | 1,8 | 66 | M | - | - | 1 | 6 | - |
| 1 | 69 | 382 | 414 | 138 | 6 | 9,5 | 3,6 | 137 | 3,4 | 9,4 | 2,2 | 82 | M | - | + | 2 | 2 | - |
| 1 | 70 | 444 | 444 | 138 | 4,9 | 8,1 | 2,4 | 137 | 3 | 8,5 | 2,1 | 79 | M |  |  | 1 | 2 | - |
| 2 | 71 | 431 | 506 | 140 | 5,3 | 10,3 | 2,4 | 139 | 3,2 | 9,1 | 1,9 | 74 | M | - | + | 2 | 2 | + |
| 2 | 72 | 413 | 499 | 138 | 5 | 8,5 |  | 140 | 3,2 | 8,3 | 2,1 | 77 | M | - | + | 1 | 2 | + |
| 2 | 73 | 424 | 471 | 140 | 5,6 | 9,2 | 2,8 | 138 | 3,2 | 9 | 2,1 | 80 | F | + | + | 1 | 1 | - |
| 2 | 74 | 450 | 465 | 134 | 4,6 | 8,9 | 3,6 | 138 | 3,3 | 8,5 | 2,5 | 56 | M | + | + | 1 | 2 | + |
| 2 | 75 | 459 | 500 | 137 | 5,6 | 7,7 | 2,2 | 140 | 2,7 | 8,8 | 2,2 | 70 | M | + | + | 2 | 1 | + |
| 2 | 76 | 440 | 626 | 139 | 5,1 | 9 | 2,6 | 141 | 3 | 9 | 2,2 | 82 | M | - | + | 2 | 2 | + |
| 2 | 77 | 450 | 516 | 140 | 3,8 | 8,1 | 2,1 | 139 | 2,9 | 8,6 | 2,1 | 69 | M | - | + | 1 | 2 | + |
| 2 | 78 | 424 | 480 | 143 | 6,1 | 7,5 | 2,2 | 140 | 3 | 9,4 | 2,1 | 57 | M | - | + | 1 | 5 | + |
| 2 | 79 | 422 | 461 | 142 | 4,9 | 8,3 | 2,7 | 140 | 3,3 | 9,5 | 2,3 | 67 | M | - | - | 1 | 3 | + |
| 2 | 80 | 377 | 462 | 138 | 5,6 | 8,8 | 2,6 | 139 |  | 9,1 | 2,1 | 61 | F | + | - | 1 | 1 | + |
| 2 | 81 | 437 | 500 | 141 | 4,8 | 7,2 | 2,3 | 141 | 2,9 | 8,3 | 2,2 | 55 | F | - | + | 2 | 7 | + |
| 2 | 82 | 454 | 514 | 135 | 4,4 | 7,5 | 2,4 | 143 | 2,6 | 8 | 2 | 78 | M | - | + | 2 | 6 | + |
| 2 | 83 | 453 | 474 | 146 | 3,9 | 8,6 | 2,4 | 140 | 3,2 | 9,1 | 2,3 | 73 | M | + | - | 2 | 2 | + |
| 2 | 84 | 420 | 480 | 141 | 4,2 | 9,7 | 2,4 | 145 | 4,1 | 9,3 | 2,5 | 44 | M | + | + | 1 | 1 | + |
| 2 | 85 | 458 | 555 | 141 | 4,5 | 8,5 | 2,6 | 142 | 3,1 | 8,8 | 2,2 | 72 | M | + | + | 2 | 1 | + |
| 2 | 86 | 429 | 469 | 137 | 5,9 | 8,1 | 2,1 | 139 | 3,4 | 8,8 | 2 | 87 | F |  |  | 1 | 2 | + |
| 2 | 87 | 437 | 506 | 140 | 3,7 | 9,4 | 2,2 | 140 | 2,7 | 8,9 | 2,1 | 73 | M |  |  | 2 | 3 | + |
| 2 | 88 | 443 | 478 | 141 | 3,9 | 8,7 | 2,4 | 138 | 2,9 | 8,8 | 2,1 | 57 | M |  |  | 1 | 1 | + |
| 2 | 89 | 413 | 499 | 140 | 4,4 | 8,9 | 2,7 | 138 | 3,1 | 8,7 | 2,3 | 77 | M | + | + | 1 | 1 | + |
| 2 | 90 | 445 | 463 | 138 | 5,4 | 8,1 | 2,2 | 136 | 3,2 | 8,7 | 2 | 83 | M | - | - | 1 | 2 | + |
| 2 | 91 | 452 | 483 | 139 | 5,7 | 9,8 | 2,2 | 141 | 2,8 | 9,3 | 1,9 | 51 | F | - | + | 1 | 3 | + |
| 2 | 92 | 450 | 494 | 134 | 3,4 | 8,4 | 2,1 | 135 | 2,6 | 9,9 | 2 | 80 | M | - | - | 1 | 1 | + |
| 2 | 93 | 404 | 483 | 140 | 5,7 | 7,6 | 1,9 | 135 | 3,1 | 8,6 | 1,8 | 33 | F | - | - | 1 | 3 | + |
| 2 | 94 | 460 | 461 | 135 | 6,1 | 10,1 | 2,8 | 135 | 2,9 | 8,9 | 2 | 80 | F | - | + | 2 | 2 | + |
| 3 | 95 | 485 | 459 | 140 | 6,1 | 8 | 2,4 | 139 | 3,2 | 8,4 | 2 | 78 | M | - | + | 1 | 3 | + |
| 3 | 96 | 499 | 413 | 141 | 4,2 | 9,1 | 2,5 | 139 | 3,1 | 9 | 2,2 | 74 | M |  |  | 2 | 5 | + |
| 3 | 97 | 480 | 449 | 139 | 5,4 | 6,6 | 2,6 | 136 | 3,5 | 8,1 | 2,3 | 62 | M | - | + | 1 | 3 | + |
| 3 | 98 | 471 | 459 | 139 | 2,6 | 9,2 | 1,9 |  |  |  |  | 71 | M | - | + | 2 | 7 | + |
| 3 | 99 | 476 | 392 | 140 | 4,8 | 7,8 | 2,8 | 136 | 2,9 | 9 | 2,3 | 52 | M | - | - | 2 | 3 | + |
| 3 | 100 | 494 | 459 | 131 | 4,2 | 8,7 | 2,1 | 137 | 2,7 | 10,7 | 2 | 66 | M | + | + | 2 | 2 | - |
| 3 | 101 | 483 | 428 | 135 | 4,2 | 8,4 | 2,5 | 136 | 2,9 | 9,4 | 1,9 | 72 | M | + | + | 2 | 1 | - |
| 3 | 102 | 463 | 413 | 135 | 4,3 | 9,4 | 2 | 135 | 2,5 | 10 | 1,8 | 84 | F | - | + | 1 | 3 | + |
| 4 | 103 | 465 | 481 | 137 | 4,8 | 8,8 | 2,6 | 138 | 2,9 | 9,6 | 2,3 | 74 | M | - | + | 2 | 5 | + |
| 4 | 104 | 481 | 462 | 137 | 4,3 | 8,9 | 3 | 137 | 3,1 | 9 | 2,2 | 68 | M | - | + | 1 | 2 | + |
| 4 | 105 | 481 | 481 | 132 | 7,4 | 9,2 | 3,4 | 139 | 3,2 | 9,5 | 2,4 | 56 | F | + | + | 2 | 1 | + |
| 4 | 106 | 475 | 506 | 141 | 5,7 | 8,4 | 2,8 | 141 | 3,7 | 8,8 | 2,3 | 83 | M | - | + | 1 | 2 | + |
| 4 | 107 | 463 | 505 | 140 | 4,1 | 7,8 | 1,5 | 139 | 2,8 | 8,9 | 1,7 | 82 | M | - | + | 2 | 2 | + |
| 4 | 108 | 476 | 523 | 137 | 4,6 | 8,1 | 1,7 | 136 | 2,6 | 8,8 | 1,7 | 74 | M | - | + | 2 | 4 | + |
| 4 | 109 | 520 | 494 | 137 | 5,2 | 7 | 2 | 135 | 3,2 | 8 | 1,9 | 67 | F | - | + | 2 | 4 | + |
| 4 | 110 | 486 | 492 | 138 | 5,3 | 8,1 | 2 | 140 | 3,8 | 8,2 | 1,8 | 79 | F | + | + | 2 | 1 | + |
| 4 | 111 | 480 | 480 | 142 | 5,2 | 8,9 | 2,6 | 139 | 3,1 | 9,7 | 1,9 | 79 | M | - | + | 1 | 3 | + |

**Abbreviations**: Group 1: Normal QTc value pre- and post-dialysis (NNLQTc); Group 2: Normal pre- but long QTc post-dialysis (NLQTc); Group 3: long QTc pre- and Normal QTc post-dialysis (LNQTc); Group 4: long QTc both pre- and post-dialysis (LQTc); QT values are shown in milliseconds. Age: expressed in years; M: male; F: female; Etiology 1: systemic; Etiology 2: vascular; Etiology 3: glomerular; Etiology 4: congenital; Etiology 5: interstitial; Etiology 6: other; Etiology 7: unknown. Empty rows indicate unavailable data. LVEF: left ventricular ejection fraction.
