# Supplementary material for: Role of genetic and electrolyte abnormalities in prolonged QTc interval and sudden cardiac death in end-stage renal disease patients
Source: PLoS One. 2018 Jul 18;13(7):e0200756. doi: 10.1371/journal.pone.0200756 (PMC6051653; doi:10.1371/journal.pone.0200756)
Supplement: S2 Table — *P value<0.05. (DOCX) [file pone.0200756.s002.docx]

**SUPPLEMENTAL MATERIAL**

**S2 Table:** Electrolyte levels of the entire cohort and the sequenced groups. *****P value<0.05.

| 111 dialysis patients | | |
| --- | --- | --- |
| Stage of dialysis | **Electrolyte** | ***P*-Value** |
| Pre-HD | Sodium | 0.414 |
|  | Potassium | 0.418 |
|  | Calcium | 0.525 |
|  | Magnesium | 0.653 |
| Post-HD | Sodium | 0.017* |
|  | Potassium | 0.67 |
|  | Calcium | 0.547 |
|  | Magnesium | 0.399 |
| 47 patients sequenced by NGS | | |
| Stage of dialysis | **Electrolyte** | ***P*-Value** |
| Pre-HD | Sodium | 0.432 |
|  | Potassium | 0.636 |
|  | Calcium | 0.808 |
|  | Magnesium | 0.097 |
| Post-HD | Sodium | 0.060 |
|  | Potassium | 0.864 |
|  | Calcium | 0.993 |
|  | Magnesium | 0.219 |
