# Supplementary material for: Role of genetic and electrolyte abnormalities in prolonged QTc interval and sudden cardiac death in end-stage renal disease patients
Source: PLoS One. 2018 Jul 18;13(7):e0200756. doi: 10.1371/journal.pone.0200756 (PMC6051653; doi:10.1371/journal.pone.0200756)
Supplement: S3 Table — (DOCX) [file pone.0200756.s003.docx]

**SUPPLEMENTAL MATERIAL**

**S3 Table.** Classification and duration in the study of the 111 studied cases.

| **Group** | **Index Case** | **Start Date** | **EndDate** | **Status** |
| --- | --- | --- | --- | --- |
| 1 | 1 | 15/05/2008 | 31/12/2014 | 1 |
| 1 | 2 | 17/04/2007 | 12/05/2013 | 2 |
| 1 | 3 | 22/09/2006 | 31/12/2014 | 1 |
| 1 | 4 | 04/12/2007 | 31/12/2014 | 1 |
| 1 | 5 | 28/07/2009 | 20/03/2013 | 2 |
| 1 | 6 | 17/05/2006 | 23/06/2013 | 2 |
| 1 | 7 | 14/08/2009 | 31/12/2014 | 1 |
| 1 | 8 | 27/09/2007 | 21/09/2013 | 2 |
| 1 | 9 | 26/10/2009 | 31/12/2014 | 1 |
| 1 | 10 | 18/04/2007 | 31/12/2014 | 1 |
| 1 | 11 | 14/01/2008 | 31/12/2014 | 1 |
| 1 | 12 | 11/08/2003 | 31/12/2014 | 1 |
| 1 | 13 | 13/05/2010 | 31/12/2014 | 1 |
| 1 | 14 | 03/08/2004 | 08/09/2013 | 3 |
| 1 | 15 | 05/11/2009 | 31/12/2014 | 1 |
| 1 | 16 | 03/03/1999 | 11/03/2014 | 3 |
| 1 | 17 | 30/05/2005 | 31/12/2014 | 1 |
| 1 | 18 | 25/02/2011 | 14/02/2013 | 3 |
| 1 | 19 | 17/01/2011 | 31/12/2014 | 1 |
| 1 | 20 | 10/08/2006 | 31/12/2014 | 1 |
| 1 | 21 | 27/10/2009 | 31/12/2014 | 1 |
| 1 | 22 | 06/06/2011 | 31/12/2014 | 1 |
| 1 | 23 | 21/11/2010 | 31/12/2014 | 1 |
| 1 | 24 | 14/02/2011 | 21/07/2014 | 2 |
| 1 | 25 | 04/11/2010 | 24/01/2014 | 2 |
| 1 | 26 | 23/07/2004 | 06/06/2005 | 1 |
| 1 | 27 | 08/12/2010 | 31/12/2014 | 1 |
| 1 | 28 | 22/04/2010 | 31/12/2014 | 1 |
| 1 | 29 | 18/10/2010 | 31/12/2014 | 1 |
| 1 | 30 | 01/05/2008 | 15/06/2014 | 3 |
| 1 | 31 | 28/06/2011 | 28/07/2014 | 2 |
| 1 | 32 | 16/09/2010 | 31/12/2014 | 1 |
| 1 | 33 | 09/11/2011 | 10/04/2013 | 3 |
| 1 | 34 | 30/07/2007 | 31/12/2014 | 1 |
| 1 | 35 | 30/04/2010 | 29/11/2012 | 2 |
| 1 | 36 | 08/01/2009 | 31/12/2014 | 1 |
| 1 | 37 | 19/08/2010 | 31/12/2014 | 1 |
| 1 | 38 | 02/11/2009 | 14/02/2013 | 2 |
| 1 | 39 | 19/09/2011 | 02/09/2014 | 3 |
| 1 | 40 | 03/05/2009 | 31/12/2014 | 1 |
| 1 | 41 | 15/01/2010 | 23/01/2013 | 3 |
| 1 | 42 | 25/01/2008 | 31/12/2014 | 1 |
| 1 | 43 | 21/02/2008 | 31/12/2014 | 1 |
| 1 | 44 | 04/11/2011 | 31/12/2014 | 1 |
| 1 | 45 | 24/03/2003 | 31/12/2014 | 1 |
| 1 | 46 | 30/12/2011 | 31/12/2014 | 1 |
| 1 | 47 | 04/08/2011 | 31/12/2014 | 1 |
| 1 | 48 | 26/12/2011 | 31/12/2014 | 1 |
| 1 | 49 | 02/12/2011 | 24/04/2013 | 3 |
| 1 | 50 | 09/01/2012 | 31/12/2014 | 1 |
| 1 | 51 | 07/02/2012 | 31/12/2014 | 1 |
| 1 | 52 | 13/03/2012 | 31/12/2014 | 1 |
| 1 | 53 | 18/01/2012 | 31/12/2014 | 1 |
| 1 | 54 | 14/02/2012 | 31/12/2014 | 1 |
| 1 | 55 | 20/02/2012 | 31/12/2014 | 1 |
| 1 | 56 | 04/03/1999 | 16/12/2014 | 2 |
| 1 | 57 | 05/07/1999 | 31/12/2014 | 1 |
| 1 | 58 | 17/05/2010 | 24/02/2014 | 3 |
| 1 | 59 | 22/04/2004 | 31/12/2014 | 1 |
| 1 | 60 | 27/06/2006 | 31/12/2014 | 1 |
| 1 | 61 | 25/03/2010 | 08/01/2014 | 2 |
| 1 | 62 | 04/08/2010 | 31/12/2014 | 1 |
| 1 | 63 | 22/09/2008 | 10/04/2013 | 2 |
| 1 | 64 | 16/11/2009 | 31/12/2014 | 1 |
| 1 | 65 | 03/08/2010 | 25/02/2013 | 2 |
| 1 | 66 | 03/03/2011 | 31/12/2014 | 1 |
| 1 | 67 | 20/04/2011 | 13/02/2013 | 3 |
| 1 | 68 | 19/12/2010 | 31/12/2014 | 1 |
| 1 | 69 | 09/03/2010 | 21/11/2013 | 2 |
| 1 | 70 | 09/07/2004 | 31/12/2014 | 1 |
| 2 | 71 | 18/02/2008 | 26/12/2014 | 2 |
| 2 | 72 | 07/11/2005 | 31/12/2014 | 1 |
| 2 | 73 | 12/04/2007 | 31/12/2014 | 1 |
| 2 | 74 | 25/05/2007 | 31/12/2014 | 1 |
| 2 | 75 | 09/01/2009 | 10/11/2013 | 2 |
| 2 | 76 | 16/04/2009 | 18/03/2013 | 2 |
| 2 | 77 | 05/01/2007 | 31/12/2014 | 1 |
| 2 | 78 | 07/06/2001 | 31/12/2014 | 1 |
| 2 | 79 | 07/10/2004 | 31/12/2014 | 1 |
| 2 | 80 | 16/02/2007 | 31/12/2014 | 1 |
| 2 | 81 | 16/07/2010 | 19/01/2014 | 2 |
| 2 | 82 | 21/04/2009 | 30/11/2014 | 2 |
| 2 | 83 | 02/11/2010 | 22/01/2013 | 2 |
| 2 | 84 | 29/08/2011 | 31/12/2014 | 1 |
| 2 | 85 | 08/08/2008 | 17/02/2014 | 2 |
| 2 | 86 | 29/12/2009 | 31/12/2014 | 1 |
| 2 | 87 | 22/09/2006 | 15/02/2014 | 2 |
| 2 | 88 | 30/12/2011 | 31/12/2014 | 1 |
| 2 | 89 | 16/02/2012 | 31/12/2014 | 1 |
| 2 | 90 | 19/06/2006 | 31/12/2014 | 1 |
| 2 | 91 | 14/07/2001 | 31/12/2014 | 1 |
| 2 | 92 | 27/01/2010 | 31/12/2014 | 1 |
| 2 | 93 | 03/05/1999 | 31/12/2014 | 1 |
| 2 | 94 | 08/02/2010 | 20/10/2011 | 2 |
| 3 | 95 | 27/03/2007 | 31/12/2014 | 1 |
| 3 | 96 | 21/09/2005 | 15/10/2013 | 2 |
| 3 | 97 | 23/08/2004 | 31/12/2014 | 1 |
| 3 | 98 | 09/09/2011 | 16/08/2013 | 2 |
| 3 | 99 | 04/12/2006 | 18/03/2013 | 2 |
| 3 | 100 | 22/11/2010 | 18/12/2012 | 2 |
| 3 | 101 | 28/09/2010 | 01/01/2014 | 2 |
| 3 | 102 | 27/12/2004 | 31/12/2014 | 1 |
| 4 | 103 | 28/11/2002 | 19/05/2014 | 2 |
| 4 | 104 | 15/04/2011 | 31/12/2014 | 1 |
| 4 | 105 | 16/06/2011 | 21/06/2014 | 2 |
| 4 | 106 | 08/09/2006 | 31/12/2014 | 1 |
| 4 | 107 | 16/06/2003 | 02/09/2014 | 2 |
| 4 | 108 | 07/01/2010 | 06/11/2013 | 2 |
| 4 | 109 | 15/01/2002 | 12/09/2013 | 2 |
| 4 | 110 | 27/04/2007 | 22/04/2014 | 2 |
| 4 | 111 | 10/06/2005 | 31/12/2014 | 1 |

**Abbreviations**: Group 1: Normal QTc value pre- and post-dialysis (NNLQTc); Group 2: Normal pre- but long QTc post-dialysis (NLQTc); Group 3: long QTc pre- and Normal QTc post-dialysis (LNQTc); Group 4: long QTc both pre- and post-dialysis (LQTc). StartDate: date started the HD treatment; EndDate: date of death or end of study (31/12/2014). Status 1: 1 patient; Status 2: Death; Status 3: Transplant patient.
