# Supplementary material for: Role of genetic and electrolyte abnormalities in prolonged QTc interval and sudden cardiac death in end-stage renal disease patients
Source: PLoS One. 2018 Jul 18;13(7):e0200756. doi: 10.1371/journal.pone.0200756 (PMC6051653; doi:10.1371/journal.pone.0200756)
Supplement: S4 Table — (DOCX) [file pone.0200756.s004.docx]

**SUPPLEMENTAL MATERIAL**

**S4 Table.** QTc values and electrolyte levels of the entire cohort for patients who were living and deceased at the end of the study.

|  | **Alive** | **Death** | ***P*-Value** |
| --- | --- | --- | --- |
| QTc pre | 417.99 (410.42-425.56) | 444.29 (431.08-457.51) | 0.000* |
| QTc post | 432.09 (424.29-439.9) | 460.06 (441.19-478.93) | 0.01* |
| Sodium pre | 139.13 (138.47-139.79) | 138.09 (137.01-139.17) | 0.089 |
| Sodium post | 139 (138.58-139.42) | 138.55 (137.81-139.28) | 0.26 |
| Potassium pre | 4.88 (4.69-5.07) | 5.04 (4.69-5.4) | 0.374 |
| Potassium post | 3.09 (3-3.17) | 3.13 (2.99-3.28) | 0.571 |
| Calcium pre | 8.59 (8.45-8.73) | 8.62 (8.34-8.9) | 0.832 |
| Calcium post | 8.94 (8.82-9.06) | 8.95 (8.74-9.16) | 0.943 |
| Magnesium pre | 2.5 (2.41-2.6) | 2.4 (2.25-2.56) | 0.269 |
| Magnesium post | 2.13 (2.09-2.17) | 2.08 (2.01-2.15) | 0.186 |
|  |  |  |  |

Sodium and potassium levels are expressed in mEq/L and calcium and magnesium levels are expressed in mg/gL. Data are presented as mean (95% CI). Results of a T-test for significant differences between groups are shown in the right column.
