# Supplementary material for: Role of genetic and electrolyte abnormalities in prolonged QTc interval and sudden cardiac death in end-stage renal disease patients
Source: PLoS One. 2018 Jul 18;13(7):e0200756. doi: 10.1371/journal.pone.0200756 (PMC6051653; doi:10.1371/journal.pone.0200756)
Supplement: S5 Table — (DOCX) [file pone.0200756.s005.docx]

**SUPPLEMENTAL MATERIAL**

**S5 Table.** Variants of uncertain significance

| **Index case** | **Gene** | **Isoform** | **SNP** | **Location** | **Type of variant** | **cDNA change** | **Alelle Frequency**** |
| --- | --- | --- | --- | --- | --- | --- | --- |
| **NNQTc** | | | | | | | |
| 10 | *DSP* | NM_004415 | rs17133512 | Exonic-Frameshit | Indel | c.-1_1insA | 0.1994 |
|  | *DSP* | NM_004415 |  | Exonic-Frameshit | Indel | c.245insC | NA |
| 11 | *DSP* | NM_004415 | rs17133512 | Exonic-Frameshit | Indel | c.-1_1insA | 0.1994 |
| 12 | *CACNA1C* | NM_001129827 |  | Exonic-Missense | SNP | c.1640A>G* | NA |
| 14 | *JUP* | NM_002230 | rs570878629  (CM098198) | Exonic-Missense | SNP | c.56C>T* | 0.0001139 |
| 44 | *DSP* | NM_004415 | rs17133512 | Exonic-Frameshit | Indel | c.-1_1insA | 0.1994 |
| 47 | *CACNB2* | NM_201596 | rs578106427 | Exonic-Missense | SNP | c.1124C>T | 1.648e-05 |
| **NLQTc** | | | | | | | |
| 72 | *CASQ2* | NM_001232 | rs28730716  (CM106915) | Exonic-Missense | SNP | c.731A>G* | 0.008292 |
|  | *SCN5A* | NM_198056 | rs6791924 | Exonic-Missense | SNP | c.100C>T* | 0.009462 |
| 74 | *DSP* | NM_004415 | rs17133512 | Exonic-Frameshit | Indel | c.-1_1insA | 0.1994 |
| 78 | *DSP* | NM_004415 | rs17133512 | Exonic-Frameshit | Indel | c.-1_1insA | 0.1994 |
| 92 | *DSP* | NM_004415 | rs17133512 | Exonic-Frameshit | Indel | c.-1_1insA | 0.1994 |
| 83 | *DSP* | NM_004415 | rs17133512 | Exonic-Frameshit | Indel | c.-1_1insA | 0.1994 |
| **LNQTc** | | | | | | | |
| 97 | *CASQ2* | NM_001232 | rs28730716  (CM106915) | Exonic-Missense | SNP | c.731A>G* | 0.008292 |
| 102 | *RYR2* | NM_001035 | rs200070226 | Exonic-Missense | SNP | c.4747C>T* | NA |
|  | *DSP* | NM_004415 | rs17133512 | Exonic-Frameshit | Indel | c.-1_1insA | 0.1994 |
| **LLQTc** | | | | | | | |
| 107 | *DSP* | NM_004415 | rs78652302 | Exonic-Missense | SNP | c.5498A>T* | 0.00946 |
|  | *DSP* | NM_004415 | rs17133512 | Exonic-Frameshit | Indel | c.-1_1insA | 0.1994 |
| 104 | *DSP* | NM_004415 | rs17133512 | Exonic-Frameshit | Indel | c.-1_1insA | 0.1994 |
| 105 | *CACNA1C* | NM_001129827 |  | Exonic-Missense | SNP | c.3428G>A* | NA |

*Provean deleterious

** Allele frequency obtained from ExAC browser. Last consulted in May 2018.

NA: not available
